# Evaluation of the MenACWY Vaccination Catch-Up Campaign Among Adolescents in Madrid: Coverage, Trends, and Determinants

**DOI:** 10.3390/vaccines14020152

**Published:** 2026-02-04

**Authors:** Pablo Estrella-Porter, Amaya Sánchez-Gómez, María Dolores Lasheras Carbajo, Patricia Guillem Sáiz, Carmen Sáiz-Sánchez, Juan José Carreras

**Affiliations:** 1 Área de Prevención de la Enfermedad, Subdirección General de Promoción, Prevención y Educación para la Salud, Dirección General de Salud Pública, Consejería de Sanidad, 28002 Madrid, Spain; 2 Department of Preventative Medicine and Public Health, Food Sciences, Toxicology and Legal Medicine, University of Valencia, 46010 Valencia, Spain; 3 Vaccine Research Department, Foundation for the Promotion of Health and Biomedical Research in the Valencian Region (FISABIO-Public Health), 46020 Valencia, Spain; juanjo.carreras@fisabio.es; 4 Biomedical Research Consortium of Epidemiology and Public Health (CIBER-ESP), Instituto de Salud Carlos III, 28029 Madrid, Spain

**Keywords:** MenACWY vaccine, adolescent immunization, meningococcus, catch-up vaccination campaign, public health, vaccine coverage

## Abstract

**Background**: Invasive meningococcal disease (IMD) caused by *Neisseria meningitidis* remains a major public health concern due to its severity, lethality, and long-term sequelae. To address the rise in serogroups W and Y in Spain, the Community of Madrid implemented a catch-up campaign in 2019–2021, targeting adolescents (ages 13–18) alongside routine tetravalent meningococcus vaccine (MenACWY) at age 12. This study evaluated MenACWY catch-up vaccination uptake in routine practice by describing vaccine coverage, temporal trends, and associated factors in adolescents born between 2001 and 2006. **Methods**: A population-based cross-sectional study was conducted using data from the Community of Madrid’s vaccination registry (SISPAL Vacunas). Vaccination coverage was calculated for adolescents with at least one recorded MenACWY dose from age 10 onwards. Temporal trends were analyzed by birth cohort and calendar time, and multivariable logistic regression models were used to identify factors associated with vaccination uptake. **Results**: Among 424,059 adolescents, overall vaccination coverage by December 2021 was 63.8%, ranging from 54.4% to 78.2% across birth cohorts. Coverage was highest in the 2006 cohort, likely due to co-administration with the tetanus and diphtheria (Td) booster. A slightly higher uptake was observed among females and adolescents with chronic conditions, while foreign-born adolescents consistently showed lower coverage. COVID-19 disruptions led to temporal variability, with sharp declines during lockdowns and partial recoveries thereafter, with persistent sociodemographic differences in uptake. **Conclusions**: By December 2021, coverage was incomplete, with marked variability across birth cohorts. Higher uptake was observed when vaccination was integrated into routine visits, while persistent sociodemographic disparities remained evident. These observational findings are consistent with the programmatic value of combined catch-up and routine strategies and the need for targeted actions to ensure equitable MenACWY coverage.

## 1. Introduction

*Neisseria meningitidis* is a diplococcus with 12 serogroups, of which six (A, B, C, W, Y, and X) are responsible for invasive meningococcal disease (IMD) [1]. This bacterium colonizes the nasopharynx and may cause infection or remain asymptomatic, particularly in adolescents and young adults, playing a central role in transmission dynamics [2]. Clinically, IMD most frequently presents as meningitis or sepsis, conditions that may rapidly progress to shock and other severe complications [3]. Transmission occurs through respiratory secretions during close or prolonged contact with carriers or infected individuals [4].

In Spain, IMD is a notifiable disease through the National Epidemiological Surveillance Network (RENAVE) and is analyzed by epidemiological seasons from week 41 to week 40 of the following year [5]. In Europe, an increase in IMD cases emerged in 2009, initially in the United Kingdom and later in the Netherlands, linked to a hypervirulent clonal complex 11 (CC11) serogroup W strain, prompting preventive strategies across the region [1,6]. In Spain, the incidence declined from the 1999–2000 season to the 2013–2014 season, after which this trend stalled and cases due to serogroups W and Y increased, reaching incidence rates of 0.16/100,000 and 0.10/100,000, respectively, by week 32 of the 2019 season [7,8]. In the 2017–2018 season, overall IMD lethality increased to 12.7%, with serogroup W showing the highest lethality (29.2%) followed by serogroup C (20.0%), while serogroup B remained lower (7.7%) [5]. Despite its relatively low incidence, IMD continues to be a public health concern in Spain due to its severity, potential complications, and long-term consequences [1].

The meningococcal vaccination strategy in Spain has evolved significantly over the last two decades. Following the introduction of the monovalent serogroup C conjugate (MenC) vaccine in 2000 for infants with a three-doses schedule, and in 2005 it changed to two doses at 2–4 months and a booster at 12 months. By 2014, the primary dose was recommended at 4 months, with boosters at 12 months and 12 years, as well as a catch-up strategy for cohorts born in 2000–2002. In 2018, the Interterritorial Council of the National Health System of Spain (CISNS) recommended the administration of one dose of MenC to adolescents up to 18 years who had not received one after 10 years of age [9]. These efforts achieved high coverage, with 96% for primary vaccination at 6 months of life in 2023 and over 70% for the 12-year booster since 2014, maintaining low IMD rates caused by serogroup C [8,10,11]. However, the rise in serogroups W and Y in Europe prompted a shift in strategy in 2019 where the tetravalent vaccine (MenACWY) replaced MenC in the routine 12-year schedule, accompanied by a national catch-up campaign targeting adolescents aged 13–18 between 2019 and 2021 [5]. This aimed to directly protect high-transmission groups and provide indirect protection to other populations, building on evidence from similar campaigns that demonstrated reduced IMD incidence through high coverage and community immunity [12,13]. Beyond the national context, meningococcal vaccination is a key global public health intervention to reduce IMD morbidity and mortality, especially in settings at risk of epidemics.

In the Community of Madrid, the meningococcal vaccination strategy was modified in 2019 following recommendations by the CISNS. MenACWY replaced MenC vaccine for 12-year-olds (born after 24 July 2007), and a catch-up campaign was launched targeting adolescents born between 2001 and 2007, regardless of previous MenC vaccination provided there was a 4-week interval from the last dose. Adolescents aged 10 years or older who had already been vaccinated with MenACWY were excluded from the campaign [5,14]. The strategy combined systematic vaccination at 12 years with a three-year catch-up period (2019–2021) and, from January 2020 onwards, incorporated MenACWY co-administration with the routine tetanus and diphtheria (Td) booster dose at 14 years. Ongoing vaccination recommendations for high-risk groups, including individuals with immunodeficiencies, HIV, or a history of IMD, were maintained to ensure broader protection [14]. The MenACWY vaccine used during this period was Nimenrix^®^ (Pfizer, New York, NY, USA) and was administered intramuscularly in the deltoid muscle [15]. This vaccine was distributed, upon demand, to all authorized vaccination centers in the Community of Madrid throughout the study period. Active recruitment strategies were implemented through primary care services, supported by an electronic application that enabled nursing staff to identify eligible adolescents by birth cohort and manage appointments at the center level [16]. Additionally, all other authorized vaccination centers in the Community of Madrid, both public and private, received doses upon demand of the MenACWY vaccine for vaccinating the indicated cohorts.

From the Health Department of the Community of Madrid, training sessions for healthcare professionals were conducted, and support materials were developed (information sheets, technical documents, frequently asked questions). Additionally, the General Directorate of Public Health, Quality, and Innovation of the Ministry of Health created an informative website in 2019 aimed at parents, adolescents, and healthcare personnel, along with infographics for these populations [17].

This study aims to evaluate the MenACWY catch-up vaccination campaign implemented in the Community of Madrid (2019–2021) by assessing vaccination coverage, temporal patterns, and demographic and socioeconomic factors associated with vaccine uptake among adolescents.

## 2. Materials and Methods

### 2.1. Study Design

A population-based cross-sectional study was conducted to evaluate the MenACWY vaccination catch-up campaign implemented in the Community of Madrid. The study population included adolescents born between 2001 and 2006 (13–18 years old), targeted for the MenACWY catch-up vaccination campaign from 24 July 2019 to 31 December 2021, the period when the campaign was performed. The primary study outcomes were MenACWY vaccination uptake, temporal trends in coverage, and factors influencing uptake. Adolescents who had received any MenACWY vaccine dose prior to turning 10 years old were excluded from the analysis of vaccine coverage.

### 2.2. Data Sources

Vaccination data were obtained from the Vaccination Registry of the Community of Madrid (SISPAL Vacunas), a mandatory nominal registry that collects information regarding all vaccines administered in both public and private healthcare settings [18]. This registry was used to identify MenACWY doses administered during the study period. The Population Information System of the Community of Madrid was used as the basic sociodemographic data record for users with a Health Card of the Madrid Public Health System [19]. Data from these sources were linked by the individual unique identifier and subsequently anonymized prior to analysis, ensuring compliance with data protection regulations.

### 2.3. Study Variables

The following covariates were considered: sex, birth cohort, country of origin, socioeconomic level, place of residence (urban or rural), and presence of chronic disease. Socioeconomic levels were assessed using the 2011 Deprivation Index from the Spanish Society of Epidemiology (IP2011), which evaluates six socioeconomic indicators (e.g., unemployment rate, manual labor, lack of internet access) and categorizes basic health zones into quintiles, with the first quintile representing the highest socioeconomic level [20].

Urban or rural residence was classified based on population density criteria from 2021 (municipalities with <30,000 inhabitants and <100 persons/km^2^ were classified as rural) [21]. Additionally, chronic disease status was identified using International Classification of Primary Care (ICPC) codes registered in the database. We considered chronic diseases when the corresponding codes from the following diseases group were registered: pulmonary diseases, cardiovascular diseases, chronic inflammatory disease, immunosuppression, neurological disease, nephrological and hepatic disease, chronic anemia, coagulopathies, and type 1 diabetes mellitus.

### 2.4. Data and Statistical Analysis

The mid-year population database was used for each campaign year to match the corresponding birth cohorts (e.g., July 2019 for those born in 2001–2002 and June 2020 for those born in 2003–2004).

Descriptive statistics were used to summarize study variables by birth cohort. Vaccination coverage was calculated for individuals with at least one recorded MenACWY dose from age 10 until the end of the study. Temporal vaccine coverage trends were analyzed based on the month and year of the first MenACWY dose.

For the analysis of factors associated with MenACWY vaccination, adolescents who had received a dose of MenACWY prior to the start of the catch-up were excluded, provided they had received it when they were at least 10 years old. Logistic regression models were applied to identify factors associated with vaccination uptake, using Odds Ratios (OR) and Adjusted Odds Ratios (aOR) with 95% confidence intervals. Due to conceptual overlap with area-level deprivation and to avoid instability from correlated area-level covariates, we presented the primary adjusted model without the residence variable. Residence was retained in bivariate analyses.

All statistical analyses were performed using IBM^®^ SPSS^®^ Statistics, Version 25.0.

## 3. Results

### 3.1. Study Population

The study population comprised 424,059 adolescents born between 2001 and 2006, with cohort sizes ranging from 66,459 (2001) to 73,384 (2004) (Table 1). Overall, 51.1% were male and 85.2% were born in Spain. The majority of adolescents (83.8%) resided in urban areas and 16.9% had a chronic disease, with chronic respiratory diseases being the most frequent, followed by anemia and cardiovascular diseases. Distribution by socioeconomic quintile was balanced with 23.5% in the highest quintile (Q1) and 17.0% in the lowest (Q5).

### 3.2. MenACWY Vaccination Coverage by Birth Cohort

By 31 December 2021, overall MenACWY vaccination coverage (≥1 dose administered from age 10 years) among adolescents included in the six catch-up cohorts (2001–2006) was 63.8% (Table 2). Coverage varied substantially by birth cohort, ranging from 54.4% in the 2005 cohort to 78.2% in the 2006 cohort. The 2001 and 2002 cohorts showed relatively higher coverage rates of 67.4% and 65.8%, respectively. Conversely, the 2003 and 2004 cohorts exhibited lower uptake at 59.2% and 58.1%.

### 3.3. Temporal Trends in MenACWY Vaccination (2019–2021)

**Figure 1:**
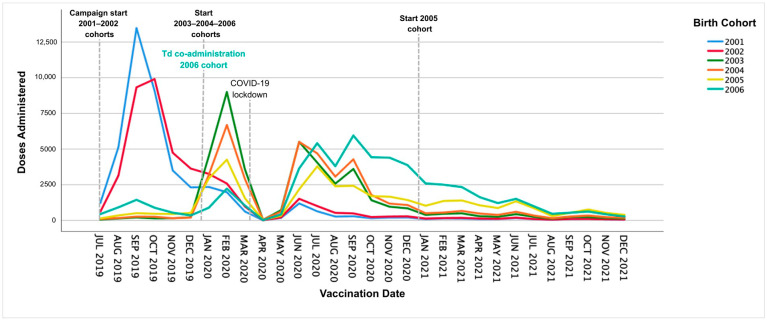
MenACWY vaccine catch-up doses administered by birth cohort between July 2019 and December 2021 in the Community of Madrid. Td: tetanus and diphtheria.

Temporal trends in MenACWY vaccine administration differed by calendar year and birth cohort (Figure 1). In 2019, the 2001 and 2002 cohorts experienced a rapid increase in vaccination rates during the initial phase of the campaign, peaking at 13,474 doses in September 2019 and 9984 doses in October 2019, respectively. In 2020, eligibility expanded to cohorts 2003, 2004, and 2006 (as part of the Td booster dose), producing additional early year peaks in February 2020 (2003 cohort: 8987 doses; 2004 cohort: 6673 doses), followed by a sharp decline in April–May 2020 during the start of the COVID-19 pandemic. In June 2020, vaccination rates rebounded primarily in the 2005 and 2006 cohorts, reaching a secondary peak in September 2020 (2006 cohort: 5950 doses).

However, the vaccination pace in the second half of 2020 did not reach the levels observed in 2019. During 2021, vaccination rates continued to decrease steadily across all cohorts. From August 2021 onward, the overall number of administered doses remained low with minimal differences between cohorts. Interpretation of these cohort-specific changes should consider the temporal overlap between the start of Td co-administration (January 2020) and subsequent COVID-19-related service disruptions.

### 3.4. Factors Associated with MenACWY Catch-Up Vaccination

Factors associated with MenACWY vaccination uptake during the catch-up campaign were analyzed after excluding adolescents who had received the vaccine prior to the start of the campaign (N = 7633, 2.8% of the total). In the bivariate analysis, a significant association was found for all studied variables. Vaccination uptake was higher among females (OR = 1.08, 95% CI = 1.07–1.09) and in adolescents with chronic diseases (OR = 1.64, 95% CI = 1.61–1.67) compared to those without chronic conditions. Individuals in intermediate socioeconomic quintiles had a higher probability of vaccination compared to those in the lowest quintile (e.g., Q3: OR = 1.25, 95% CI = 1.23–1.28), and coverage was also lower among individuals born abroad (OR = 0.35, 95% CI = 0.34–0.35).

In the multivariable analysis, “residential area” was not included in the primary adjusted model due to overlap with area-level deprivation and to improve model stability; adjusted associations for the remaining covariates are shown in Table 3. It was observed that females continued to show a higher likelihood of vaccination (aOR = 1.11, 95% CI = 1.09–1.12), as did adolescents with chronic diseases (aOR = 1.49, 95% CI = 1.47–1.52) and those from lower socioeconomic quintiles. The 2006 cohort had a significantly higher probability of vaccination compared to the 2001 cohort (aOR = 1.60, 95% CI = 1.56–1.64), while the 2005 cohort showed the lowest likelihood (aOR = 0.51, 95% CI = 0.50–0.52).

## 4. Discussion

This population-based study provides a comprehensive evaluation of the MenACWY adolescent catch-up vaccination campaign implemented in the Community of Madrid between July 2019 and December 2021. By the end of this campaign period, 63.8% of adolescents born between 2001 and 2006 (270,631 target individuals) had received at least one dose of MenACWY, with substantial heterogeneity by birth cohort and sociodemographic characteristics. Vaccination uptake was higher among females, adolescents with chronic conditions, and those residing in intermediate socioeconomic strata, whereas markedly lower coverage was observed among foreign-born adolescents. The vaccine administration’s temporal progression followed a clear sequence by calendar year: older cohorts (born 2001–2002) were prioritized in 2019, eligibility expanded to 2003, 2004, and 2006 in 2020, and residual catch-up continued in 2021 when pandemic-related disruptions tempered monthly volumes but not the overall gains. Overall, these temporal trends reflected both the planned programmatic prioritization by birth cohort and the subsequent disruption caused by the COVID-19 pandemic.

The overall coverage achieved in the Community of Madrid compares favorably with that reported in several countries implementing similar adolescent catch-up programs, such as the UK, where coverage of young adults aged 19 to 22 varied between 36.7% and 40.8% in 2019 [22]. However, vaccination coverage in the Community of Madrid in 2021 was lower when comparing the coverage of recruitment campaigns in school or university settings carried out in other countries in these environments at similar time periods. In England, even during the onset of the COVID-19 pandemic, vaccination coverage above 75% was maintained in cohorts targeted in schools from 2020 to 2021 [23]. A similar situation occurred in the Netherlands, where the estimated vaccination coverage in adolescents was around 85% among the birth cohorts from 2001 to 2005 in the years 2018 to 2020 [24]. However, vaccination campaigns in educational settings presented other logistical and ethical challenges [25].

The notably high coverage observed in the 2006 birth cohort (78.2%) was likely attributable to the implementation of the co-administration of MenACWY with the routine Td booster vaccine introduced in January 2020 [15]. Co-administration strategies have consistently been effective in other countries, where integrating MenACWY-TT into national programs significantly reduced disease incidence (83–85%) in target cohorts, such as in Chile, England, and Australia, while also providing indirect protection to non-vaccinated groups [26]. Therefore, enhancing vaccination opportunities through scheduled visits, ensuring timely administration, and co-administering vaccines when recommended by their technical guidelines are essential strategies to increase vaccination coverage [27,28]. Given the temporal overlap between the introduction of MenACWY–Td co-administration and COVID-19-related changes in healthcare delivery and health-seeking behavior, these findings should be interpreted as programmatic associations rather than evidence of a direct causal effect.

In 2019, the catch-up program primarily targeted the 2001 and 2002 birth cohorts. The highest monthly peaks of the entire study period happened in September 2019 (2001 cohort: 13,474 doses) and October 2019 (2002 cohort: 9984 doses). In early 2020, eligibility was extended to include the 2003, 2004, and 2006 birth cohorts, resulting in further peaks in the early months of the year, particularly in February 2020, with 8987 and 6673 doses administered to the 2003 and 2004 cohorts, respectively. The number of monthly doses administered fell sharply during the April–May 2020 lockdown due to the COVID-19 pandemic, then rebounded from June 2020. A secondary peak was reached in September 2020 for the 2006 cohort (5950 doses), although vaccine administration rates in late 2020 did not return to those seen in 2019. In 2021, residual catch-up was concentrated in the 2005 and 2006 birth cohorts, with local peaks in January 2021 (2639 doses for the 2006 cohort) and March 2021 (1407 doses for the 2005 cohort). However, the monthly doses administered were lower than in the previous two years, reflecting the ongoing impact of the pandemic on vaccination services [29]. In the 2019–2020 season, Spain saw a 31% decrease in IMD cases compared to 2018–2019 [30], followed by a rise in IMD incidence from 2020 to 2021 through week 38 of 2024 [31]. This initial reduction likely resulted from COVID-19 control measures, such as physical distancing, lockdowns, masks, and hygiene, that also hindered *Neisseria meningitidis* transmission [24]. Furthermore, the pandemic could have impacted meningitis surveillance and diagnosis systems, resulting in underreporting [30,32].

Importantly, the post-lockdown rebound remained incomplete, with vaccination volumes in late 2020 and 2021 not returning to the initial 2019 campaign intensity. This likely reflected a combination of sustained health-system constraints and demand-side factors after the acute pandemic phase (e.g., reduced opportunistic contacts, competing priorities, and the inherent difficulty of achieving high uptake in adolescent programs without systematic delivery channels). Because we did not measure communication intensity or reminder/recall activity, we cannot determine which specific operational components drove the observed deceleration. Furthermore, the absence of harmonized, comparable time-series data on MenACWY catch-up implementation across Spanish regions and European countries limited a formal comparison.

Female sex was associated with slightly higher odds of vaccination (aOR = 1.11), although the absolute difference in coverage was modest (64.0% vs. 62.2%). Given the large population size, small relative differences are expected to reach statistical significance; therefore, this association should be interpreted cautiously and primarily in terms of programmatic relevance rather than statistical significance alone [33]. Evidence on sex differences in vaccine uptake is mixed, and we interpret this finding cautiously [34,35]. Potential explanations can include small differences in health-service utilization and preventive-care engagement during adolescence, or unmeasured social factors influencing appointment adherence [36]; however, we did not measure these mechanisms and cannot draw causal inferences.

Other key social factors have been identified to have an impact on MenACWY vaccination uptake. A lower probability was observed in foreign-born individuals compared to those born in Spain (aOR = 0.35; *p* < 0.001), potentially reflecting disparities in healthcare access, cultural factors, and socioeconomic conditions [37,38]. A U-shaped pattern was noted for socioeconomic levels, with both the highest (Q1) and lowest (Q4, Q5) levels associated with lower uptake, possibly due to varying perceptions of risk and healthcare accessibility [39,40]. Adolescents with chronic conditions showed increased vaccination rates (aOR = 1.49; *p* < 0.001), likely due to heightened perceived risk and closer healthcare contact [41].

The use of administrative databases to evaluate vaccine coverage has many strengths, but also some limitations. Although SISPAL Vacunas is a mandatory nominal registry, we could not directly quantify completeness or timeliness of reporting by provider type; differential recording between public and private settings could lead to under-ascertainment of doses in specific subgroups. There may be underreporting in the vaccination registry or changes in the residence of the adolescent population without them being unregistered from the Health Card database. While additional contextual annotations may help interpretation, the absence of harmonized, comparable data across regions and countries precluded a formal comparative visualization.

The country-of-origin variable grouped all births outside Spain into a single category, lacking length-of-residence, language- or nationality-specific detail. This prevented the underlying associated factors in this category from being analyzed in more detail. Other factors to take into consideration can include misclassification, like in chronic conditions derived from routine ICPC coding which may be under-recorded and do not capture severity, and would typically bias associations toward the null if these were non-differential. Similarly, deprivation is assigned at the basic health-zone level and may not reflect individual socioeconomic position, leaving residual confounding within quintiles. Finally, the study period overlapped with COVID-19 service disruptions and the introduction of MenACWY co-administration with Td (in the 2006 birth cohort), limiting the study’s ability to distinguish campaign effects from concurrent system changes.

This study’s main strengths included its large, population-based design encompassing 424,059 adolescents using a mandatory, individual-level vaccination registry linked to comprehensive sociodemographic data system, enabling the analysis of temporal trends during the COVID-19 pandemic and the lockdown and showing how health-system disruptions and policy adaptations influenced vaccination rates. Nevertheless, several limitations merit consideration. As with any administrative database, underreporting of administered doses or changes in the residence of the adolescent population without them being unregistered from the Health Card database cannot be ruled out. Population mobility and delayed updating of Health Card registrations may disproportionately affect foreign-born adolescents, which could contribute to denominator inflation or missed-dose capture in this subgroup. Because ‘foreign-born’ aggregates heterogeneous trajectories (e.g., recent arrivals vs. long-term residents), this finding should be interpreted as a marker of inequity rather than a direct measure of specific mechanisms. Future analyses linking vaccination uptake to proxies of health-systems or more granular country/region-of-origin and language variables would help identify the most actionable barriers post-lockdown rebound that remain incomplete.

Important health-system factors that may have influenced catch-up success, such as variability in patient outreach, outreach success, staffing, or center-level processes, were not evaluated. Such unmeasured implementation heterogeneity could plausibly contribute to the differences observed across cohorts and socioeconomic strata.

Vaccination in adolescents has historically represented a challenge in achieving similar coverage to that observed in early childhood immunization programs [42]. Despite the progress made during the first 3 years of the MenACWY catch-up program, more than one-third of the target adolescent population remained unvaccinated by the end of 2021. Recent reports published in 2024 have shown the increase in vaccination coverage of MenACWY vaccine in the adolescent population of the Community of Madrid, with over 90% coverage in the 12-year cohort by the year 2023 [43]. Nevertheless, the “3Cs model” is useful for understanding factors contributing to low uptake, focusing on three key elements: confidence (trust in vaccine effectiveness and safety), complacency (low perceived risk of infection), and convenience (availability and accessibility of vaccination services) [44]. Additionally, it is important to develop strategies that take into account the use of other communication channels, such as social media or active recruitment in educational centers, which can contribute to increasing coverage [45]. Addressing these barriers comprehensively is essential for developing effective campaigns and improving adolescent vaccination rates.

Meningococcal disease remains a significant public health threat, and adolescent MenACWY vaccination represents a cornerstone of prevention strategies [46]. In the Community of Madrid, targeted catch-up MenACWY campaigns for those up to 18 years old increased coverage from 63.8% in 2021 (this study) to 91.7% by 2023 in the corresponding 2010 birth cohort, underscoring the long-term effectiveness of these vaccine strategies [27,43]. It is important to note the need for an efficient surveillance system for the early detection of changes in IMD incidence and serogroups to act promptly in vaccination strategies [47].

## 5. Conclusions

The MenACWY adolescent catch-up vaccination campaign implemented in the Community of Madrid achieved substantial, yet incomplete, coverage by the end of 2021. Coverage levels were below those typically required for sustained indirect protection, underscoring the need for complementary strategies to achieve long-term, population-level impact, with marked heterogeneity across birth cohorts. Higher uptake was observed in cohorts benefiting from integration into routine vaccination visits and co-administration with the Td booster, underscoring the importance of embedding catch-up strategies within established healthcare contacts. Given the role of adolescents in meningococcal carriage and transmission, the remaining unvaccinated proportion may limit the potential for sustained indirect protection, reinforcing the need for additional strategies to raise and equalize uptake.

Vaccination uptake was uneven across sociodemographic groups, with a slightly higher uptake observed among females though the absolute differences were small, higher coverage among adolescents with chronic conditions, and those residing in intermediate socioeconomic strata, while there was a persistently lower uptake among foreign-born adolescents. These findings highlight the need for equity-focused interventions and tailored outreach strategies to address structural- and access-related barriers in adolescent immunization programs.

Overall, these observational findings were consistent with the programmatic value in combining catch-up activities with routine vaccination opportunities to increase MenACWY uptake. Sustained integration into routine schedules alongside targeted strategies to reach underserved groups are needed to improve coverage and reduce inequities.

## Figures and Tables

**Table 1:** Description of the population of adolescents born between 2001 and 2006 with records in the healthcare card database, Community of Madrid.

		2001 Cohort (N = 66,459)		2002 Cohort (N = 68,763)		2003 Cohort (N = 71,763)		2004 Cohort (N = 73,384)		2005 Cohort (N = 71,037)		2006 Cohort (N = 72,653)	
Variables		N	%	N	%	N	%	N	%	N	%	N	%
**Sex**	**Males**	33,812	50.9%	35,202	51.2%	36,671	51.1%	37,790	50.9%	36,215	50.9%	37,171	50.9%
	**Females**	32,647	49.1%	33,561	48.8%	35,092	48.9%	35,594	49.1%	34,822	49.1%	35,482	49.1%
**Country of origin**	**Spain**	53,551	80.6%	56,970	82.8%	60,494	84.3%	62,982	85.8%	62,309	87.7%	65,033	89.5%
	**Other**	12,908	19.4%	11,793	17.2%	11,269	15.7%	10,402	14.2%	8728	12.3%	7620	10.5%
**Socioeconomic level (Quintile)**	**Q1**	15,188	22.9%	16,017	23.3%	16,930	23.6%	17,154	23.4%	16,971	23.9%	17,228	23.7%
	**Q2**	13,099	19.8%	13,554	19.8%	14,178	19.8%	14,275	19.5%	13,815	19.5%	14,031	19.3%
	**Q3**	15,771	23.8%	15,921	23.2%	16,760	23.4%	17,135	23.4%	16,424	23.2%	16,567	22.8%
	**Q4**	11,177	16.9%	11,426	16.7%	11,724	16.4%	12,134	16.6%	11,664	16.5%	12,098	16.7%
	**Q5**	11,089	16.7%	11,700	17.1%	12,059	16.8%	12,550	17.1%	12,022	17.0%	12,617	17.4%
**Place of residency**	**Rural**	10,256	15.4%	10,749	15.6%	11,362	15.8%	11,564	15.8%	11,560	16.3%	11,782	16.2%
	**Urban**	56,203	84.6%	58,014	84.4%	60,401	84.2%	61,820	84.2%	59,477	83.7%	60,871	83.8%
**Chronic disease**	**No**	55,156	83.0%	57,044	83.0%	59,573	83.0%	60,921	83.0%	58,909	82.9%	60,859	83.8%
	**Yes**	11,303	17.0%	11,719	17.0%	12,190	17.0%	12,463	17.0%	12,128	17.1%	11,794	16.2%

**Table 2:** Vaccination coverages against Meningococcus ACWY in target catch-up adolescent cohorts born between 2001 and 2006, Community of Madrid in 2021.

	Vaccine Coverage	
Cohort	N	%
**2001**	44,812	67.4%
**2002**	45,224	65.8%
**2003**	42,488	59.2%
**2004**	42,605	58.1%
**2005**	38,670	54.4%
**2006**	56,832	78.2%
**Total**	270,631	63.8%

**Table 3:** Factors associated with MenACWY vaccination during the catch-up in individuals born between 2001 and 2006, Community of Madrid during the period 2019–2021.

Variables		% Vaccinated	Bivariate Analysis OR (95% CI)	Multivariate Analysis aOR (95% CI)
**Cohort**	**2001**	66.9%	(ref)	(ref)
	**2002**	65.1%	0.92 (0.90–0.95)	0.90 (0.88–0.92)
	**2003**	58.3%	0.69 (0.68–0.71)	0.65 (0.64–0.67)
	**2004**	57.0%	0.65 (0.64–0.67)	0.60 (0.59–0.62)
	**2005**	53.6%	0.57 (0.56–0.58)	0.51 (0.5–0.52)
	**2006**	77.8%	1.74 (1.69–1.78)	1.60 (1.56–1.64)
**Sex**	**Male**	62.2%	(ref)	(ref)
	**Female**	64.0%	1.08 (1.07–1.09)	1.11 (1.09–1.12)
**Country of origin**	**Spain**	66.9%	(ref)	(ref)
	**Other**	41.2%	0.35 (0.34–0.35)	0.35 (0.34–0.35)
**Socioeconomic level (Quartile)**	**Q1**	59.1%	(ref)	(ref)
	**Q2**	67.0%	1.41 (1.38–1.43)	1.46 (1.43–1.49)
	**Q3**	64.4%	1.25 (1.23–1.28)	1.32 (1.29–1.34)
	**Q4**	62.2%	1.14 (1.12–1.16)	1.24 (1.21–1.27)
	**Q5**	63.1%	1.18 (1.16–1.21)	1.31 (1.28–1.34)
**Place of residency**	**Rural**	64.7%	(ref)	-
	**Urban**	62.8%	0.92 (0.91–0.94)	-
**Chronic pathology**	**No**	61.3%	(ref)	(ref)
	**Yes**	72.1%	1.64 (1.61–1.67)	1.49 (1.47–1.52)

OR: Odds Ratio; aOR: Adjusted Odds Ratio; CI: Confidence Interval.

## Data Availability

The original contributions presented in this study are included in the article. Further inquiries can be directed to the corresponding author.

## Abbreviations

The following abbreviations are used in this manuscript:

IMD	Invasive Meningococcal Disease
MenACWY	Meningococcus serogroups A, C, W and Y (tetravalent meningococcal vaccine)
IP2011	2011 Deprivation Index from the Spanish Society of Epidemiology
ICPC	International Classification of Primary Care
Td	Tetanus and diphtheria
